# The Guanine Nucleotide Exchange Factor ARNO mediates the activation of ARF and phospholipase D by insulin

**DOI:** 10.1186/1471-2121-4-13

**Published:** 2003-09-11

**Authors:** Hai-Sheng Li, Kuntala Shome, Raúl Rojas, Megan A Rizzo, Chandrasekaran Vasudevan, Eric Fluharty, Lorraine C Santy, James E Casanova, Guillermo Romero

**Affiliations:** 1 Departments of Pharmacology, University of Pittsburgh School of Medicine, Pittsburgh, PA 15261 USA; 2 Cell Biology and Physiology, University of Pittsburgh School of Medicine, Pittsburgh, PA 15261 USA; 3 Department of Cell Biology, University of Virginia School of Medicine, Charlottesville, VA 22908 USA

**Keywords:** Insulin Receptor, Digitonin, Insulin Stimulation, Sec7 Domain, Human Insulin Receptor

## Abstract

**Background:**

Phospholipase D (PLD) is involved in many signaling pathways. In most systems, the activity of PLD is primarily regulated by the members of the ADP-Ribosylation Factor (ARF) family of GTPases, but the mechanism of activation of PLD and ARF by extracellular signals has not been fully established. Here we tested the hypothesis that ARF-guanine nucleotide exchange factors (ARF-GEFs) of the cytohesin/ARNO family mediate the activation of ARF and PLD by insulin.

**Results:**

Wild type ARNO transiently transfected in HIRcB cells was translocated to the plasma membrane in an insulin-dependent manner and promoted the translocation of ARF to the membranes. ARNO mutants: ΔCC-ARNO and CC-ARNO were partially translocated to the membranes while ΔPH-ARNO and PH-ARNO could not be translocated to the membranes. Sec7 domain mutants of ARNO did not facilitate the ARF translocation. Overexpression of wild type ARNO significantly increased insulin-stimulated PLD activity, and mutations in the Sec7 and PH domains, or deletion of the PH or CC domains inhibited the effects of insulin.

**Conclusions:**

Small ARF-GEFs of the cytohesin/ARNO family mediate the activation of ARF and PLD by the insulin receptor.

**Electronic supplementary material:**

The online version of this article (doi:10.1186/1471-2121-4-13) contains supplementary material, which is available to authorized users.

## Background

**Figure 1:**
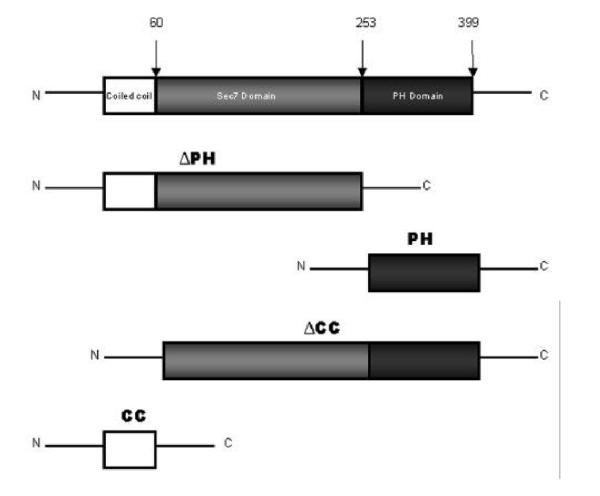
**Schematic structure of ARNO constructs.** Full length of wild type ARNO and ΔPH-ARNO were subcloned either in pCMV-myc or pEGFP-C1. PH-ARNO and ΔCC-ARNO were subcloned in pEGFP-C1. CC-ARNO was subcloned in pEGFP-N1.

Small GTPases of the ADP-ribosylation factor (ARF) family play a major role in membrane trafficking in eukaryotic cells [1]. ARF activation is facilitated by specific guanine nucleotide exchange factors (ARF-GEFs). Several ARF-GEFs have been identified, varying in size, structure and subcellular distribution [2–6]. Of particular interest in signaling events are the members of the cytohesin/ARNO family of ARF-GEFs. These proteins have been found to associate with the plasma membrane under certain conditions, and consist of three well-defined motifs: an N-terminal coiled-coil domain (CC domain), a central domain with homology to the yeast protein Sec7 (Sec7 domain), and a C-terminal pleckstrin homology domain (PH domain) (Fig. 1). The catalytic activity of ARNO for guanine nucleotide exchange is localized in the Sec7 domain and appears to be regulated through the interaction of the PH domain with phosphatidylinositol (PtdIns) (3,4,5)-P3 [7, 8], an intermediate in signaling cascades regulated by insulin and other agonists [3].

Phospholipase D (PLD) catalyzes the hydrolysis of phosphatidylcholine (PC) to produce phosphatidic acid (PA). It is involved in a variety of signaling pathways and membrane traffic processes [9, 10]. Many hormones, neurotransmitters, and growth factors, including insulin, have been shown to induce the activation of PLD [11, 12]. Several factors are involved in the regulation of cellular PLD activity, such as Ca^2+^, protein kinase C, tyrosine kinases, and G proteins [13–17]. Among these, the members of the ARF and Rho families of GTPases appear to be the most potent physiological activators [18–24]. However, the mechanism of the activation of PLD by ARF and Rho has not yet been fully established.

This study was designed to investigate the role of ARNO in the regulation of PLD activity by insulin in HIRcB cells, a Rat-1 fibroblast cell line that overexpresses human insulin receptors. The objectives were: 1) to test if insulin induces the translocation of wild type ARNO to the plasma membrane in transiently transfected HIRcB cells; 2) to determine whether ARNO translocation is accompanied by activation and subcellular translocation of ARF; 3) to explore if overexpression of wild type ARNO in HIRcB cells alters insulin-dependent PLD activity; and 4) to investigate the function of individual domains of ARNO in insulin-dependent PLD and ARF activation.

## Results

### Insulin–dependent binding of ARNO to cell membranes

**Figure 2:**
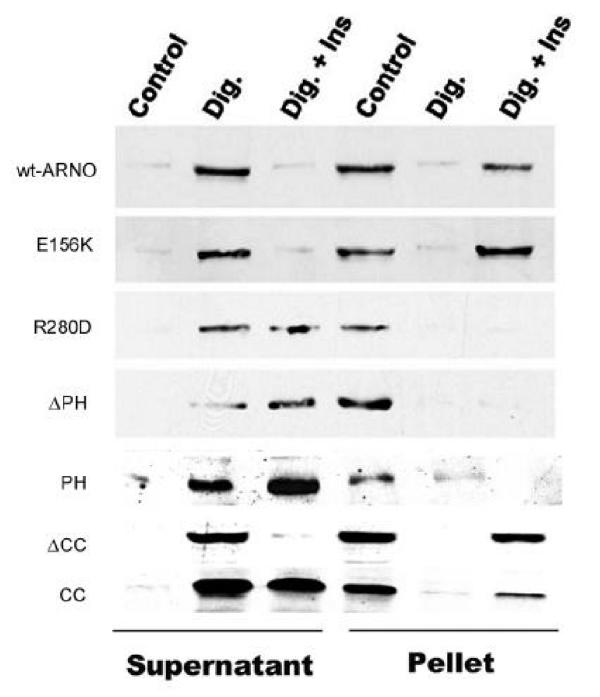
**Insulin promotes the translocation of ARNO to cell membranes.** HIRcB cells were transfected with myc-wt-ARNO, myc-E156K-ARNO, myc-R280D-ARNO, myc-ΔPH-ARNO, EGFP-PH-ARNO, EGFP-ΔCC-ARNO, and CC-ARNO-EGFP. The cells were treated with/without (Control) 10 μM digitonin (Dig). Where indicated, 100 nM insulin, 1 mM ATP, and 100 μM GTPγS were present during permeablization reaction. Pellets and supernatants were separated by centrifugation and the presence of myc-ARNO and its mutants or ARNO-EGFP in each fraction was determined by immunoblotting.

The translocation of ARNO and ARNO mutants to the membranes was studied in HIRcB cells using a digitonin permeabilization assay. For these experiments, HIRcB cells were transiently transfected with myc-tagged wild type ARNO and the following mutants: ΔPH-ARNO, PH-ARNO, ΔCC-ARNO, CC-ARNO, E156K-ARNO and R280D-ARNO. This assay is based on the formation of pores in the plasma membrane induced by digitonin to allow cytosolic proteins to leak out of treated cells upon centrifugation. Fig. 2 shows that, after digitonin permeablization, a significant fraction of ARNO proteins leaked out of serum-starved HIRcB cells that transiently overexpressed the wild type ARNO and its mutants. Since these proteins were mostly recovered from the supernatant fractions, suggesting that wild type ARNO and the mutants tested are predominantly cytosolic in non-stimulated cells. In contrast, when digitonin permeablization was performed in the presence of insulin (100 nM), most of wt-ARNO, E156K-ARNO, and ΔCC-ARNO as well as a part of CC-ARNO were recovered from the particulate membrane fraction, suggesting that these ARNO proteins can be recruited to the membrane by insulin to various degrees. However, neither R280D-ARNO nor ΔPH-ARNO was recovered from the particulate fraction after insulin stimulation, suggesting that the translocation of ARNO to the membrane requires an intact PH-domain. It should be noted that, although the CC domain alone binds to the membranes under stimulation conditions, the degree of the binding is much less than that of wild type ARNO (Fig. 2). Surprisingly, a construct containing only the PH domain of ARNO could not be recruited to the membranes by insulin, indicating that the PH domain is essential but not sufficient for the translocation of ARNO.

### ARNO recruits ARF1 to the plasma membrane in an insulin-dependent manner

**Figure 3:**
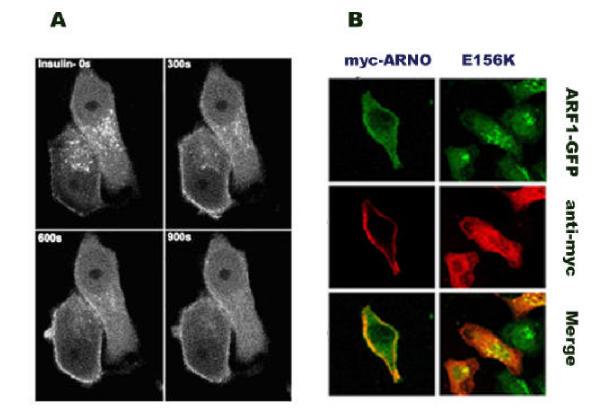
**A. Real time image of the translocation of ARF1-GFP to the plasma membrane.** HeLa cells that had been stably transfected with ARF1-GFP were transiently transfected with myc-ARNO, serum starved overnight, and treated with 100 nM insulin. Images were collected every 30 seconds using a Molecular Dynamics 2001 confocal microscope. The time intervals that were indicated on the upper right hand corner of each panel represent the time after the addition of insulin. **B. The translocation of ARF1-GFP to the plasma membrane by the effects of insulin requires ARNO.** ARF1-GFP/HeLa cells were transfected with myc-ARNO, treated, fixed, and stained for myc-epitope as described in the Materials and Methods section. Images displaying ARF1-GFP (green) and myc-ARNO (red) were merged us ing Adobe Photoshop software.

Since ARNO is an activation factor of ARF, we tested the hypothesis that agonist-dependent ARNO translocation facilitates the local binding of ARF proteins to the membrane. An initial set of real-time studies was done using HeLa cells that had been stably transfected with an ARF1-GFP construct [25]. These cells were transfected with myc-ARNO, serum-starved overnight, and imaged with a confocal microscope equipped with a constant-temperature microperfusion incubator to maintain the temperature at 37°C. Time-lapse images were collected at 30-second intervals. A representative experiment was shown in Fig. 3A. Prior to insulin stimulation, ARF1-GFP protein was mostly cytosolic or bound to the Golgi apparatus, although a small amount of ARF-GFP was localized on the surface of the cells. Ten minutes after the insulin stimulation, most of the ARF1-GFP was found on the plasma membrane. Similar results were obtained with HIRcB cells co-transfected with ARNO-myc and ARF1-GFP (Fig. 3B). It should be noted that a significant accumulation of ARF1-GFP on the plasma membrane was not observed in the cells that had not been transfected with ARNO (not shown), or that had been transfected with the inactive mutant E156K-ARNO (Fig. 3B). Since the endogenous levels of ARNO in HeLa cells were so low that the protein could not be detected in Western blots, it is reasonable to assume that under physiological conditions only a very small fraction of ARF1 translocates to the plasma membrane in response to extracellular agonists.

### ARNO interacts directly with the insulin receptor

Our previous work has shown that the insulin receptor co-immunoprecipitates with ARF in an agonist-dependent manner [23]. Furthermore, we have also shown that an ARF-GEF activity is associated with the insulin receptor and that this activity is not a function of the receptor itself [23]. Given that many receptor tyrosine kinases form complexes with their target proteins, we tested the hypothesis that ARNO binds the insulin receptor.

**Figure 4:**
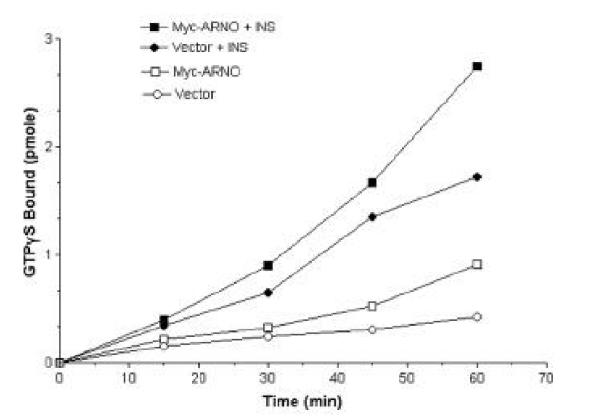
**The ARF-GDP exchange activity of the coimmunoprecipitates with the insulin receptor.** The exchange activity was determined as described in Materials and Methods. (○,□) Receptors were immunoprecipitated in the absence of insulin from cells transfected with empty vector (○) or with myc-ARNO (□). (●,■) Receptors were immunoprecipitated in the presence of insulin from cells transfected with empty vector (●) or with myc-ARNO (■).

Figure 4 shows that insulin receptors that were immunoprecipitated in the presence of insulin were associated with an ARF-GEF activity (Fig 4 ●), and that the ARF-GEF activity that was co-immunoprecipitated with the insulin receptor was significantly increased in the cells that had been transiently transfected with myc-ARNO (Fig. 4 ■). Insulin receptors that were immunoprecipitated in the absence of insulin did not accelerate the binding of GTPγS to the recombinant ARF1 as much as those obtained in the presence of insulin (Fig. 4 ○), indicating that the association of ARF-GEF activity with the insulin receptor was dependent on the presence of insulin.

**Figure 5:**
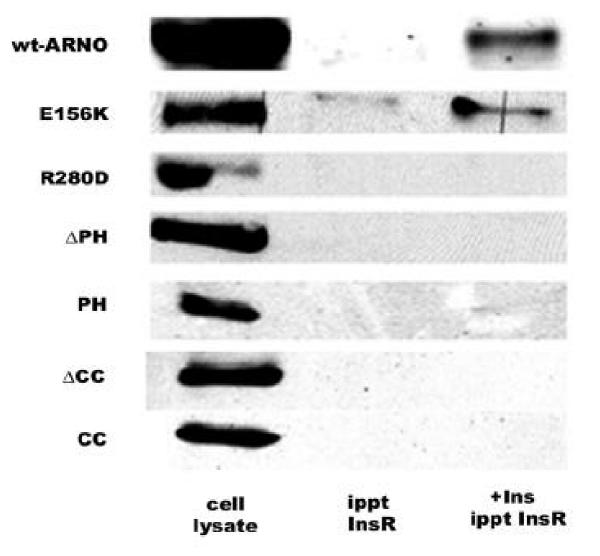
**Immunoprecipitation of the insulin receptor with ARNO and its mutants.** Immunoprecipitated proteins were resolved by SDS-PAGE and myc-ARNO, myc-E156K-ARNO, myc-R280D-ARNO and myc-ΔPH-ARNO were detected by immunoblotting with a monoclonal anti-myc epitope antibody. PH-ARNO-EGFP, ΔCC-ARNO-EGFP, and CC-ARNO-EGFP were detected by immunoblotting with a polyclonal antibody against EGFP.

We then transfected HIRcB cells with myc-tagged ARNO constructs. Fig. 5 shows that the wild type ARNO co-immunoprecipitated with the insulin receptor in an insulin-dependent manner. E156K-ARNO was also co-immunoprecipitated with the insulin receptor upon insulin stimulation. However, none of the deletion mutants, including ΔPH-ARNO, PH-ARNO, ΔCC-ARNO, and CC-ARNO, as well as a site-directed mutant R280D-ARNO, was found co-immunoprecipitated with the insulin receptor. These data suggest that ARNO directly interacts with the insulin receptor and that the interaction requires intact PH and CC domains, but the catalytic activity of the Sec7 domain does not alter the interaction.

### Effects of the overexpression of ARNO or its mutants on insulin-dependent PLD activity

We have shown so far that ARNO mediates the translocation of ARF proteins to the plasma membrane with insulin stimulation. Since ARF proteins mediate the activation of PLD by insulin [23], we tested the hypothesis that ARNO may play a role in the regulation of PLD activitiy upon insulin stimulation. To prove this point, the PLD activity of HIRcB cells that had been transiently transfected with the wild type ARNO, and mutant ARNO constructs.

**Figure 6:**
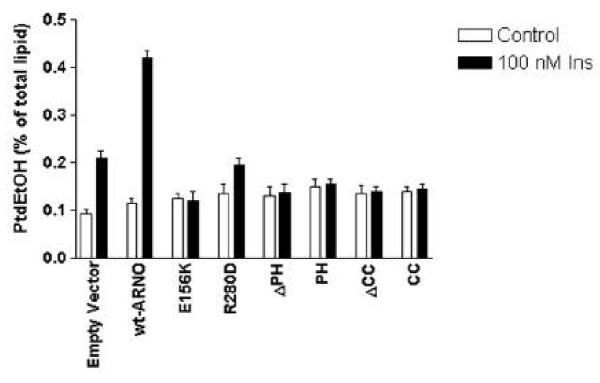
**Effects of overexpression of the wild type and mutant ARNO constructs on the activation of phospholipase D by insulin.** HIRcB cells were trans fected with empty vector, myc-wt-ARNO, myc-E156K-ARNO, myc-R280D-ARNO, and myc-ΔPH ARNO, PH-ARNO-EGFP, ΔCC-ARNO-EGFP, and CC-ARNO-EGFP. PLD activity was determined by a transphosphatidylation assay as described in Materials and Methods.

Fig. 6 shows that the overexpression of the wild type ARNO significantly increased insulin-induced PLD activity when compared with that of non-transfected cells. In contrast, the overexpression of the indicated ARNO mutants significantly decreased the ability of insulin to stimulate PLD. We conclude, therefore, that members of the cytohesin/ARNO family of ARF GEFs play an important role in the regulation of PLD activity by insulin.

## Discussion

Several studies have demonstrated that ARF proteins may mediate receptor-dependent activation of PLD. Stimulation of cell surface receptors with agonists, such as insulin, promotes the translocation of ARF proteins to the cell membranes and the activation of ARF proteins and the subsequent activation of PLD [16, 18, 21, 23]. However, the mechanisms by which ARF proteins are activated by cell surface receptors remain obscure.

ARF GEFs of the cytohesin/ARNO family have been shown to be recruited to cell membranes by mechanisms that are influenced by extracellular agonists [7, 26]. These GEFs have been implicated in the regulation of many cellular processes, ranging from the regulation of cell motility [27] to cell adhesion [28] and, more recently, oncogenesis [29]. It has been speculated that PLD activation may mediate several of the cellular events regulated by cytohesin/ARNO GEFs [30]. However, a direct proof of a role for these factors in the regulation of the receptor-mediated PLD activation is still lacking. To address these and other related issues, we have studied in detail some of the mechanistic aspects of this pathway using a fibroblast cell line that overexpresses human insulin receptors as a model. This model and other similar ones have been used in our laboratory and others to examine specific aspects of insulin receptor function, such as receptor phosphorylation and traffic [23, 31–33] and the regulation of the MAPK pathway [34].

Our studies showed that insulin promoted the translocation of myc-tagged ARNO constructs to the plasma membrane. This result is in agreement with data previously published by Venkateswarlu et al [7] and Langille et al [35] who demonstrated the insulin-dependent translocation of ARNO and the related protein GRP-1 to the plasma membrane, respectively. A detailed analysis of ARNO deletion and point mutants demonstrated that: 1) the translocation of ARNO to the membrane is independent of its ARF-GEF activity; 2) ARNO translocation to the plasma membrane requires an intact PH domain; 3) the CC domain of ARNO plays a role in targeting ARNO to the plasma membrane; 4) neither the PH domain of ARNO nor its CC domain alone sufice to target the protein to the plasma membrane; and 5) the plasma membrane translocation of ARNO is strongly regulated by insulin and, perhaps, other extracellular agonists.

The linkage between ARNO translocation to specific subcellular fractions and ARF activation was studied using myc-tagged ARNO and ARF-GFP constructs in two different cell types. Our data showed conclusively that insulin promoted the co-localization of wild type myc-ARNO and ARF1-GFP on the surface of HIRcB and HeLa cells. Interestingly, insulin, acting through ARNO, promoted the translocation of ARF1-GFP to the plasma membrane. ARF1, like most members of the ARF family, is primarily a cytosolic protein that exerts its function on specific membranes to which it is recruited by specific activators that promote the binding of GTP. However, ARF1 seems to act primarily at the Golgi, promoting the binding of coatomer proteins to the Golgi membrane [36, 37]. Nevertheless, the fact remains that ARF1 is primarily cytosolic, and that only a small fraction of it is bound to the Golgi membrane at any time [36]. It is not surprising, therefore, that some ARF1 may bind to the plasma membrane after being locally activated by ARNO, which is in turn recruited to the cell surface by the action of insulin. It should be remembered that our cells overexpress ARF1-GFP. Whether ARF1 does in fact work at the plasma membrane under physiological conditions or not remains to be established. Our data simply establish the fact that a receptor-dependent mechanism to recruit ARF1 to the plasma membrane does exist. On the other hand, ARF6 is normally found associated with the plasma membrane [36, 38], and there is evidence that ARF6 might be the primary target for ARF-GEFs of the cytohesin/ARNO family [27]. However, when ARF dominant negative mutants were tested for their ability to inhibit agonist-dependent PLD activation, the data showed that ARF1 dominant negative mutants (T31N-ARF1) were as efficient as ARF6 mutants (T27N-ARF6) [23]. These observations strongly support the idea that ARF-GEFs of the cytohesin/ARNO family have full access to the cytosolic ARF proteins. Therefore, although ARF6 might be the primary intermediate for ARNO-regulated PLD activation, other ARF proteins may as well play an important role in the pathway.

The ability of insulin to promote the translocation of ARNO and ARF to the plasma membrane correlated well with the ability of insulin to promote the activation of PLD. Therefore, our data support the hypothesis that the activation of PLD by insulin is mediated by ARF-GEFs of the cytohesin/ARNO family by a mechanism that involves the interaction of the PH and CC domains of these GEFs with some specific cellular targets. This conclusion is based on the demonstration that ARNO constructs with catalytically inactive domain or the mutants with defective PH and CC domains acted as dominant inhibitors of insulin-dependent PLD activation. The dominant negative effects of E156K-ARNO were not unexpected, since this mutant contains the intact PH and the CC domains and is therefore likely to compete with endogenous ARNO. The dominant negative effect of the PH and the CC domain deletion mutants on PLD activation was of particular interest. These mutants were at best partially translocated to the membrane but blocked the ability of insulin to promote ARF and PLD activation. This result was somewhat surprising since these deletion mutants contain an intact Sec7 domain and, therefore, would have been expected to support ARF and PLD activity. However, this was not the case, suggesting that all regions of ARNO play an important role in the regulation of this protein. Moreover, the failure of the ΔCC mutant to activate ARF and PLD indicates that other cellular targets that bind to the CC doma in of ARNO and regulate the subcellular location or the function of the signaling protein complex may exist. In fact, some proteins that interact strongly with the CC domain of members of the ARNO family, such as CASP and GRASP, have already been identified [39, 40]. Consistent with these ideas was the observation that the overexpression of either the PH or the CC domain alone was sufficient to block insulin-dependent PLD activation. Therefore, we propose that cellular targets that recognize both the PH and CC domains of ARNO are important for the regulation of the function of this protein by cell surface receptors.

On the other hand, our data also strongly support the hypothesis that the regulation of ARNO activity by insulin involves, at least transiently, a direct interaction of the insulin receptor with ARNO. Consistently, the presence of an ARNO-like activity and ARNO in the immunoprecipitated materials was confirmed by biochemical experiments. Finally, ARNO constructs lacking either the CC or the PH domain, or with a defective PH domain, failed to co-immunoprecipitate with the insulin receptor. These findings suggest a mechanism of the activation in which the binding of ARNO to the membrane is regulated by the insulin receptor at two different levels: 1) ARNO must interact with the receptor; and 2) ARNO must interact with the membrane, either via binding to polyphosphoinositides or through the interaction with specific protein targets. Our data strongly support the idea that both CC and PH domains play a crucial role in this phenomenon.

## Conclusions

This study suggests a general model for the activation of PLD with insulin stimulation. Insulin, upon binding to its receptor, promotes the phosphorylation of IRS-1 and the activation of PI3 kinase. This results in the accumulation of polyphosphoinositides on the plasma membrane. In parallel, the insulin-bound receptor promotes the recruitment of ARNO (and/or other members of the ARNO family, such as GRP-1) to the plasma membrane, either by direct interaction with their CC and PH domains or by promoting the interaction of ARNO with other as yet unidentified targets. The binding of ARF-GEFs to the plasma membrane is stabilized by the interactions of their PH domain with polyphosphoinositides generated by the action of PI3 kinase. Once on the membrane, the ARF-GEFs catalyze the activation of membrane-bound ARF6 or cytosolic ARF proteins that are then recruited to the membrane where they may activate PLD.

## Cell culture

Rat-1 fibroblasts overexpressing the human insulin receptors (HIRcB cells) were cultured in Dulbecco's modified Eagle's medium (DMEM)/Ham's F-12, supplemented with 10% fetal bovine serum, antibiotics, and 100 nM methotrexate, as previously described [20]. Cells were subcultured, transfected as indicated in the figure legends, and serum starved for overnight (approximately 20 hrs) prior to insulin stimulation.

HeLa cells were cultured in DMEM supplemented with 10% fetal bovine serum and antibiotics. HeLa-ARF1-GFP stable transfectants were obtained by using G418 as a selection agent as described elsewhere [25]. Clonal populations were obtained and used in the assays described here.

## Transient Transfection

Subconfluent (70–90%) HIRcB cells were transfected with LipofectAMINE (Invitrogen) for biochemical analyses or Superfect (QIAGEN) for imaging analyses. Transfection was performed according to the manufacturer's instructions. Transfection efficiencies were 70–90% for LipofectAMINE and 40–50% for Superfect transfection reagent as previously described [41].

## Generation of fusion proteins

It has been reported that the members of the cytohesin/ARNO family of ARF-GEFs each exist in two isoforms in terms of existence of extra G (glycine) in PH domain [42]. In this study, we used the isoform of ARNO with GGG (tri-glycine), which has similar binding affinities for both PI-(3,4,5)-P_3_ and PI-(4,5)-P_3_. The following myc-tagged ARNO constructs were generated: wt-ARNO, ΔPH-ARNO, PH-ARNO, ΔCC-ARNO, E156K-ARNO, and R250D-ARNO. wt-ARNO, ΔPH-ARNO (amino acids 1 to 269), PH-ARNO (amino acids 262–399), and ΔCC-ARNO (amino acids 51–399) (Fig. 1) were amplified by PCR and subcloned in the multiple cloning site of the vector pEGFP-C1 (CLONTECH) and fused to green fluorescent protein (GFP) as described by Venkateswarlu and coworkers [7]. The CC domain of ARNO (amino acids 1 to 55) (Fig. 1) was PCR out of wt-ARNO and subcloned into pEGFP-N1 using BglII and EcoRI restriction sites. E156K-ARNO (inactive Sec7 domain) was generated by site-directed mutagenesis as described by Frank and coworkers [43]. R280D-ARNO was designed on the basis of that a mutation on an analogous arginine impairs the binding of cytohesin-1 to polyphosphoinositides [26]. The sequences of the constructs were verified by direct sequencing and the expression of appropriate fusion proteins was examined by Western blotting. The level of expression of all constructs was found to be comparable.

## Immunoprecipitation assay

Transfected and serum-starved HIRcB cells were washed with ice-cold PBS, scraped, and collected by centrifugation. The cell pellets were solubilized on ice for 1 hr in a solution of 50 mM Hepes, pH 7.45, containing 100 mM NaCl, 1.5% sodium cholate, 1 mM EDTA, 1 mM EGTA, 5 ug/ml leupeptin, 1 mM PMSF, and 1 mg/ml soybean trypsin inhibitor. Insoluble materials were removed by centrifugation. The cell lysate was immunoprecipitated with anti-mouse IgG agarose that had been equilibrated with a monoclonal antibody 83.7 (which recognizes the α subunit of the human insulin receptor). Immunoprecipitation was carried out overnight (approximately 20 hrs) at 4°C. The immunoprecipitates were washed with lysis buffer, resuspended in SDS-PAGE sample buffer, and subjected to Western blotting analysis.

## Immunoblotting

Proteins were separated by SDS-PAGE, transferred to a nitrocellulose membrane, and blocked with 5% non-fat milk in PBS containing 0.1% Tween at room temperature for 2 hrs. The membrane was then cut in half horizontally. The upper part was used to detect the β subunit of the insulin receptor with a monoclonal antibody, CT-1, that recognizes the carboxyl terminus of the β subunit of the human insulin receptor. The lower part was used to detect ARNO proteins with a monoclonal antibody anti-myc or a polyclonal antibody anti-GFP.

## PLD activity assay

Serum-starved HIRcB cells were labeled overnight with ^3^H-palmitate (5 μCi/ml) in serum-free medium. The cells were stimulated with insulin (100 nM) in the presence of 0.5–1% ethanol for 20 min. The reaction was stopped by addition of chloroform: methanol (1:1). The lipid phase was extracted and developed by thin layer chromatography (TLC) on silica gel 60 plates using ethyl acetate: trimethylpentane: acetic acid (9: 5: 2) as a solvent. The position of major phospholipids was determined using true standards (Avanti Biochemicals) and autoradiography. The TLC plates were scraped and the total amount of radioactivity associated with each lipid species was determined by liquid scintillation counting. The data were expressed as the number of counts associated with the phosphatidylethanol (PtdEtOH) spot normalized by the total number of counts of lipid.

## Digitonin treatment

Serum-starved HIRcB cells were collected, resuspended in PBS, and treated with 10 μM digitonin in the presence or absence of insulin (100 nM), ATP (1 mM), and GTPγS (100 μM) at 37°C for 15 min. To release intracellular proteins, the digitonin-treated cells were centrifuged in a microcentrifuge for 20 min. The supernatants and the cell pellets were collected separately, and subjected to SDS-PAGE. ARNO proteins were detected by immunoblotting as described above.

## In vitro ARF activation assay

ARF activation was determined by the binding of GTPγS to the purified, myristoylated recombinant human ARF1 (mhARF1), as described by Shome and coworkers [23]. The insulin receptor was immunoprecipitated in the presence or absence of 100 nM insulin as described above. Four to 8 μg mhARF1 and the immunoprecipitated insulin receptors were incubated with 100 nM GTPγ[^35^S] (1 μCi) in 20 mM Hepes buffer containing 2 mM MgCl_2_/ 0.1% Na-cholate / 1 mM ATP. At the indicated time points, the reaction was quenched by addition of 100 μM ice-cold, unlabeled GTPγS and the protein-bound nucleotide was determined by filtration through nitrocellulose filters as described [23].

## Confocal microscopy

HIRcB cells were plated on poly-L-lysine coated glass coverslips and transfected with the constructs as indicated above. Cells were serum starved overnight and stimulated with 100 nM insulin. Live cells were imaged in a LSM5 Zeiss laser scanning confocal microscope equipped with a 63X oil immersion objective.

For ARF and ARNO colocalization experiments, HIRcB cells were plated on poly-L-lysine coated coverslips as described above and co-transfected with myc-ARNO and ARF-GFP constructs using Superfect transfection reagent according to the manufacturer's instructions. Following insulin stimulation, the cells were fixed with 4% fresh paraformaldehyde in PBS at 4°C for 30 min, and permeabilized in 0.1% Triton X-100 at room temperature for 2 min. After permeabilization, the cells were blocked with 3% bovine serum albumin in PBS at room temperature for 30 min, and immunostained with a monoclonal antibody 9E10 (Upstate Biotechnology) that recognizes the myc epitope. After extensively washing, the cells were incubated with a Cy5-conjugated donkey anti-mouse secondary antibody (Jackson Immunoresearch) and imaged using a Zeiss laser scanning confocal microscope with filters appropriate for the detection of GFP and Cy5.

## Authors’ original submitted files for images

Below are the links to the authors’ original submitted files for images. Authors’ original file for figure 1 Authors’ original file for figure 2 Authors’ original file for figure 3 Authors’ original file for figure 4 Authors’ original file for figure 5 Authors’ original file for figure 6
