# Supplementary figures and images for: The Guanine Nucleotide Exchange Factor ARNO mediates the activation of ARF and phospholipase D by insulin
Source: BMC Cell Biol. 2003 Sep 11;4:13. doi: 10.1186/1471-2121-4-13 (PMC212319; doi:10.1186/1471-2121-4-13)

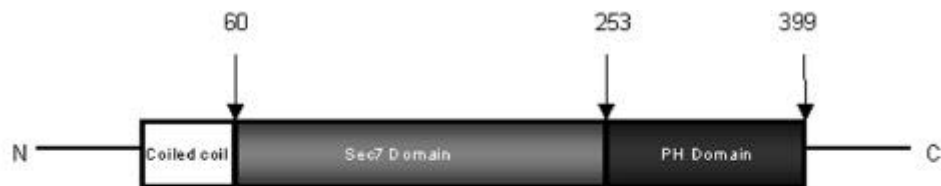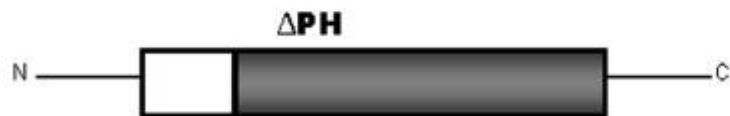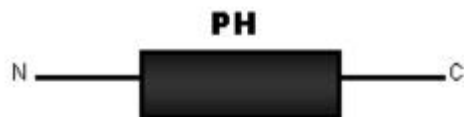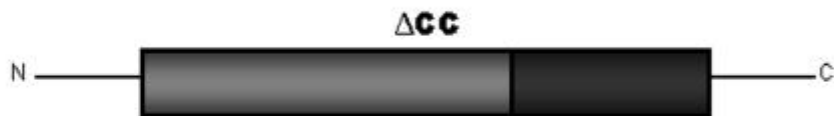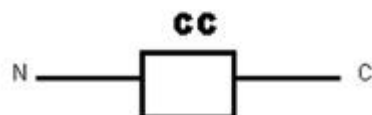

Supplement: Supplementary file 1 — Authors’ original file for figure 1 [file 12860_2003_72_MOESM1_ESM.pdf]

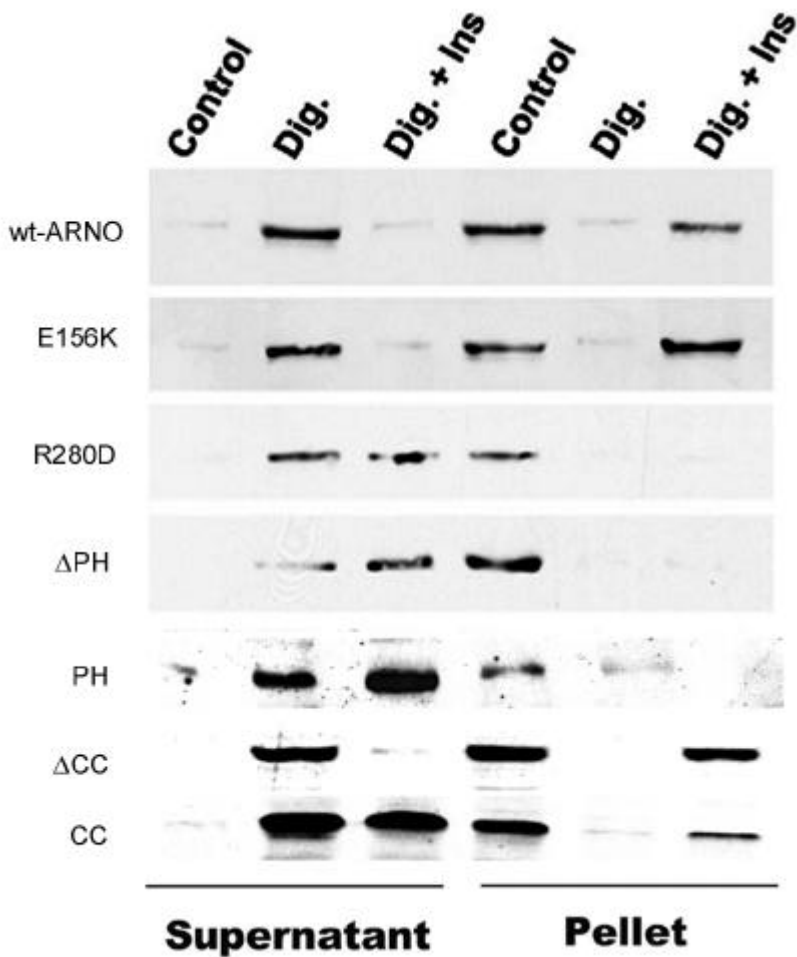

Supplement: Supplementary file 2 — Authors’ original file for figure 2 [file 12860_2003_72_MOESM2_ESM.pdf]

**A**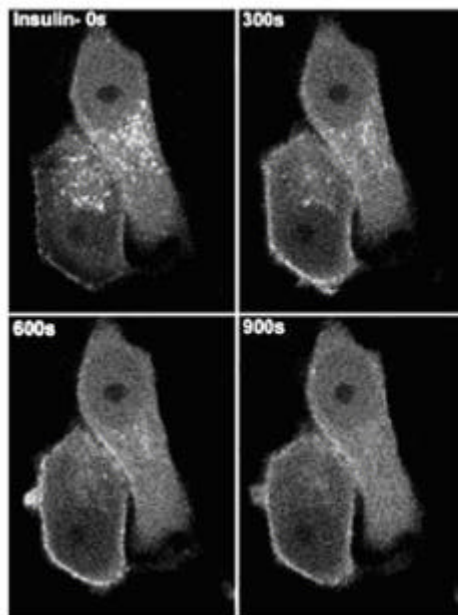**B**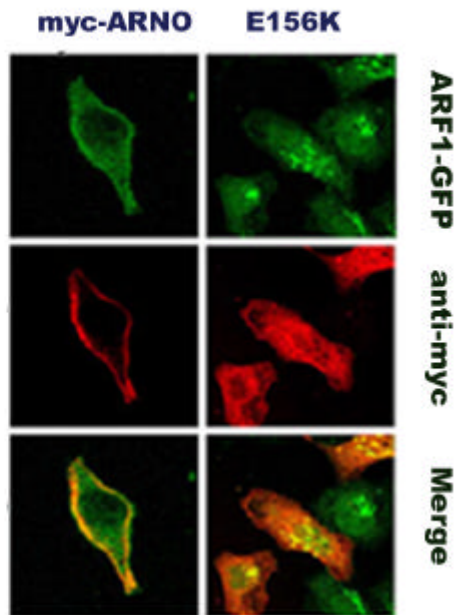

Supplement: Supplementary file 3 — Authors’ original file for figure 3 [file 12860_2003_72_MOESM3_ESM.pdf]

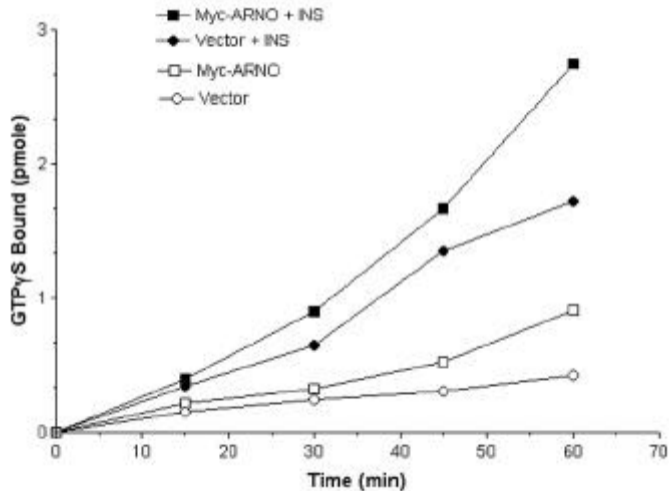

Supplement: Supplementary file 4 — Authors’ original file for figure 4 [file 12860_2003_72_MOESM4_ESM.pdf]

**wt-ARNO**

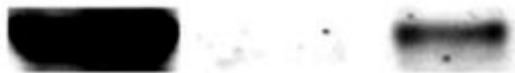

**E156K**

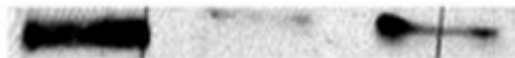

**R280D**

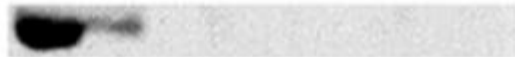

**$\Delta$ PH**

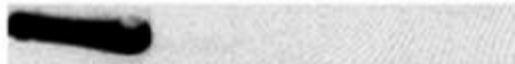

**PH**

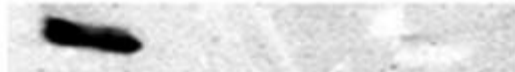

**$\Delta$ CC**

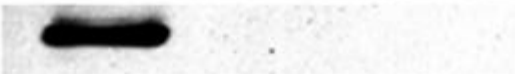

**CC**

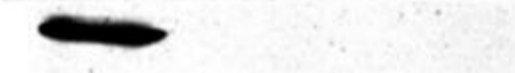

**cell  
lysate**

**ippt  
InsR**

**+Ins  
ippt InsR**

Supplement: Supplementary file 5 — Authors’ original file for figure 5 [file 12860_2003_72_MOESM5_ESM.pdf]

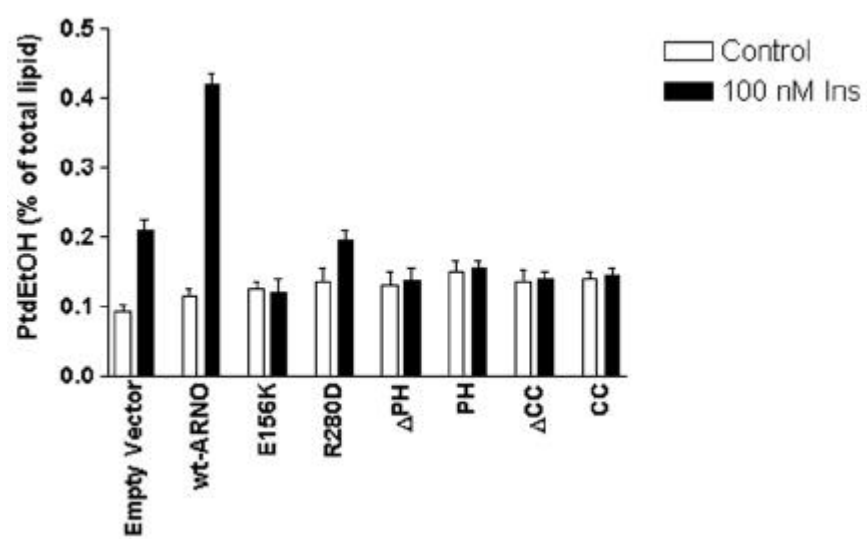

Supplement: Supplementary file 6 — Authors’ original file for figure 6 [file 12860_2003_72_MOESM6_ESM.pdf]
